# New Species of *Aspergillus* (Aspergillaceae) from Tropical Islands of China

**DOI:** 10.3390/jof8030225

**Published:** 2022-02-24

**Authors:** Xin-Cun Wang, Wen-Ying Zhuang

**Affiliations:** State Key Laboratory of Mycology, Institute of Microbiology, Chinese Academy of Sciences, Beijing 100101, China

**Keywords:** Ascomycota, Eurotiales, fungal biodiversity, phylogeny, taxonomy

## Abstract

*Aspergillus* species are cosmopolitan and ubiquitous, closely related to human daily life. They are also of food, industrial and medical importance. From the examination of cultures isolated from soil samples collected on tropical islands of China, four new species of the genus were discovered based on phylogenetic analyses and morphological comparisons. *Aspergillus xishaensis* sp. nov. and *A. neoterreus* sp. nov. belong to sections *Flavipedes* and *Terrei* of subgenus *Circumdati*, and *A. hainanicus* sp. nov. and *A. qilianyuensis* sp. nov. are in sections *Cavernicolarum* and *Nidulantes* of subgenus *Nidulantes*. To accommodate *A. hainanicus*, a new series *Hainanici* was proposed. Detailed descriptions and illustrations of the new taxa were provided.

## 1. Introduction

Species of *Aspergillus* P. Micheli ex Haller are cosmopolitan and ubiquitous. Some of them are closely related to human daily life. Strains of *A. niger* Tiegh. and *A. oryzae* (Ahlb.) Cohn were used for the fermentation of food for more than two millennia and the manufacturing of food enzymes for over 50 years [1]. *Aspergillus niger* is also a workhorse and cell factory for the production of citric acid, an organic acid with high economic importance, which is widely used in beverage, food, detergents, cosmetics and pharmaceutical industries [2]. Aflatoxins, produced by *A. flavus* Link and other aspergilli, are highly toxic secondary metabolites and severely contaminate food supplies of humans and animals, resulting in health hazards and even death [3]. Some black aspergilli were reported to be postharvest pathogens of economically important crops, e.g., *A aculeatus* Iizuka, *A. japonicus* Saito and *A. uvarum* G. Perrone et al. infecting the fruits of grapes [4]. Aspergillosis infections caused by *Aspergillus* species are of significant morbidity and mortality. Mostly, they are attributed to *A. fumigatus* Fresen., followed by *A. flavus* and *A. terreus* Thom [5].

The genus was originally introduced in 1729 and has more than one thousand names recorded in the database Index Fungorum. According to a recent monographic study, *Aspergillus* was divided into six subgenera (namely, *Aspergillus*, *Circumdati*, *Cremei*, *Fumigati*, *Nidulantes* and *Polypaecilum*), 27 sections and 75 series, with 446 species accepted [6]. Recently, more than 20 new species were added, e.g., *A. kumbius* (Pitt) and *A. malvicolor* A.D. Hocking in sect. *Circumdati*, *A. agricola* Pummi Singh et al. and *A. burnettii* Pitt in section *Flavi*, *A. alboluteus* F. Sklenar et al. and *A. okavangoensis* Visagie and Nkwe in section *Flavipedes*, *A. nanangensis* Pitt in section *Janorum*, *A. hydei* Doilom and *A. vinaceus* Ferranti et al. in section *Nigri*, and *A. barbosae* A.C.R. Barros-Correia et al. in section *Terrei* of subgenus *Circumdati*; *A. arizonensis* Jurjević et al. and *A. banksianus* Pitt in section *Fumigati* of subgenus *Fumigati*; *A. lannaensis* N. Suwannarach et al. in section *Sparsi*, and *A. sigarelli* B.D. Sun et al. in section *Usti* of subgenus *Nidulantes*; *A. limoniformis* Z.F. Zhang and L. Cai and *A. telluris* B.D. Sun et al. in sect. *Polypaecilum* of subgenus *Polypaecilum* [7,8,9,10,11,12,13,14,15,16,17,18,19]. The increasing number of species reveals the extremely high biodiversity of *Aspergillus*.

During the examinations of the cultures isolated from sandy soil collected on tropical islands of China, four new species were discovered based on phylogenetic analyses and morphological comparisons. They belong to sections *Flavipedes* and *Terrei* of subgenus *Circumdati* and sections *Cavernicolarum* and *Nidulantes* of subgenus *Nidulantes*, respectively. The detailed descriptions and illustrations of the new taxa are provided.

## 2. Materials and Methods

### 2.1. Fungal Materials

Cultures were isolated from sandy soil collected on tropical islands of China in 2015. Dried cultures were deposited in the Herbarium Mycologicum Academiae Sinicae (HMAS), and living ex-type strains were preserved in the China General Microbiological Culture Collection Center (CGMCC).

### 2.2. Morphological Observations

Morphological characterization was conducted following standardized methods [20]. Four standard growth media were used: Czapek yeast autolysate agar (CYA, yeast extract Oxoid), malt extract agar (MEA, Amresco), yeast extract agar (YES) and potato dextrose agar (PDA). If sporulation failed on the above media, PDA with 3% sea salts (3% NaCl, Psaitong) and oatmeal agar (OA) were further applied. The methods for inoculation, incubation, microscopic examinations and digital recordings followed our previous studies [21,22,23,24].

### 2.3. Molecular Experiments

DNA was extracted from the cultures grown on PDA for 7 days using the Plant Genomic DNA Kit (DP305, TIANGEN Biotech, Beijing, China). Polymerase chain reaction (PCR) amplifications of the internal transcribed spacer (ITS), beta-tubulin (BenA), calmodulin (CaM) and RNA polymerase II second largest subunit (RPB2) gene regions were conducted with the routine methods [21,22,23,24]. The products were purified and subject to sequencing on an ABI 3730 DNA Sequencer (Applied Biosystems). Although the ITS region is proposed as the universal DNA barcode for fungi, it is not sufficient to distinguish species of *Aspergillus*. The ITS sequences provided in this study might be helpful for other researchers in case of need.

### 2.4. Phylogenetic Analyses

Forward and reverse sequences newly generated in this study were assembled using Seqman v. 7.1.0 (DNASTAR Inc., Madison, WI, USA). The assembled sequences were deposited at GenBank. The sequences used for phylogenetic analyses are listed in Table 1 and Table 2. Sequences of the combined loci (BenA, CaM and RPB2) of each of the two subgenera were aligned using MAFFT v. 7.221 [25] and then manually edited and combined in BioEdit v. 7.1.10 [26] and MEGA v. 6.0.6 [27]. The combined datasets of individual subgenera were analyzed to infer their phylogeny. Maximum likelihood (ML) analyses were conducted using RAxML-HPC2 [28] on XSEDE 8.2.12 on CIPRES Science Gateway v. 3.3 [29] with the default GTRCAT model. Bayesian inference (BI) analyses were performed with MrBayes v. 3.2.5 [30]. Appropriate nucleotide substitution models and parameters were determined by Modeltest v. 3.7 [31]. The consensus trees were viewed in FigTree v. 1.3.1 (http://tree.bio.ed.ac.uk/software/figtree/ (accessed on 3 June 2015). *Aspergillus flavus* of subgen. *Circumdati* sect. *Flavi* served as an outgroup.

## 3. Results

### 3.1. Phylogenetic Analysis

**Figure 1:**
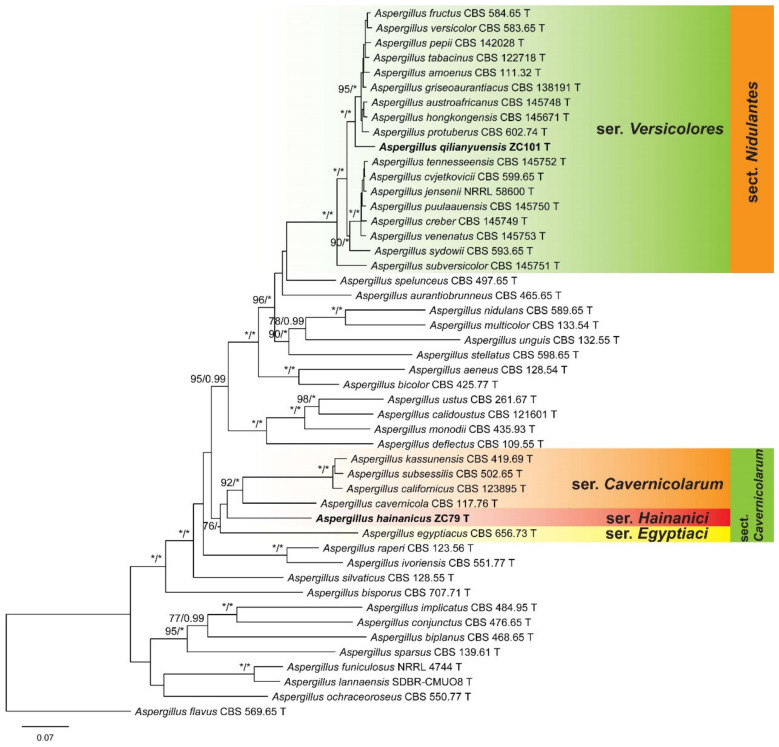
ML phylogeny of *Aspergillus* subgen. *Nidulantes* inferred from combined BenA, CaM and RPB2 dataset. Bootstrap values ≥70% (**left**) or posterior probability values ≥0.95 (**right**) are indicated at nodes. *Asterisk denotes 100% bootstrap or 1.00 posterior probability.

**Figure 2:**
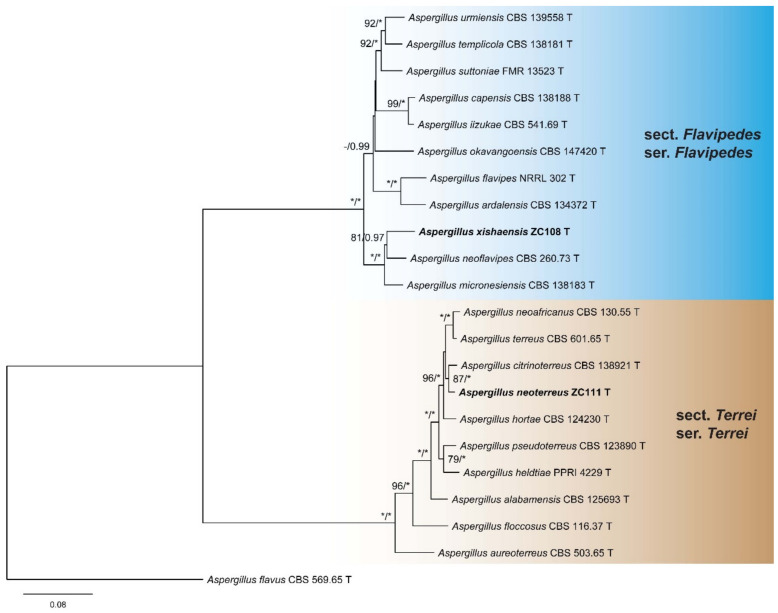
ML phylogeny of *Aspergillus* subgen. *Circumdati* inferred from combined BenA, CaM and RPB2 dataset. Bootstrap values ≥70% (**left**) or posterior probability values ≥0.95 (**right**) are indicated at nodes. *Asterisk denotes 100% bootstrap or 1.00 posterior probability.

To determine the positions of the isolates, two combined datasets (BenA + CaM + RPB2) of *Aspergillus* subgenera *Nidulantes* and *Circumdati* were compiled and analyzed. The detailed characteristics of the datasets are listed in Table 3. In the phylogeny of *Aspergillus* subg. *Nidulantes* (Figure 1), the strains ZC79 and ZC101 were located in sect. *Cavernicolarum* and *Nidulantes*, respectively. The strain ZC79 was sister to the species of ser. *Cavernicolarum* and *Egyptiaci*, and a new series was proposed as ser. *Hainanici* to accommodate it. The strain ZC 101 formed a distinct lineage in ser. *Versicolores*. As shown in the phylogenetic tree of *Aspergillus* subg. *Circumdati* (Figure 2), the strain ZC108 was a member of sect. *Flavipedes* ser. *Flavipedes*, and clustered with *A. micronesiensis* and *A. neoflavipes*. The strain ZC111 was revealed to be affiliated to sect. *Terrei* ser. *Terrei*, and shared a close relationship with *A. citrinoterreus*.

### 3.2. Taxonomy

**Series *Hainanici*** X.C. Wang and W.Y. Zhuang, ser. nov.

Fungal Names: FN570966.

**Etymology**: Named after *Aspergillus hainanicus*.

**Type**: *Aspergillus hainanicus* X.C. Wang and W.Y. Zhuang.

In Aspergillus subgen. Nidulantes sect. Cavernicolarum.

**Diagnosis**: Series *Hainanici* belongs to subgen. *Nidulantes* sect. *Cavernicolarum* and is sister to series *Cavernicolarum* and *Egyptiaci* (Figure 1). Colonies no growth at 37 °C; conidia *en masse* greyish black; conidiophores biseriate; stipes short, thick walls, brown; vesicles globose to subglobose; metulae cylindrical to obovate, covering almost a half surface of the vesicle; phialides flask-shaped; conidia large, subglobose, strongly echinulate.

**Figure 3:**
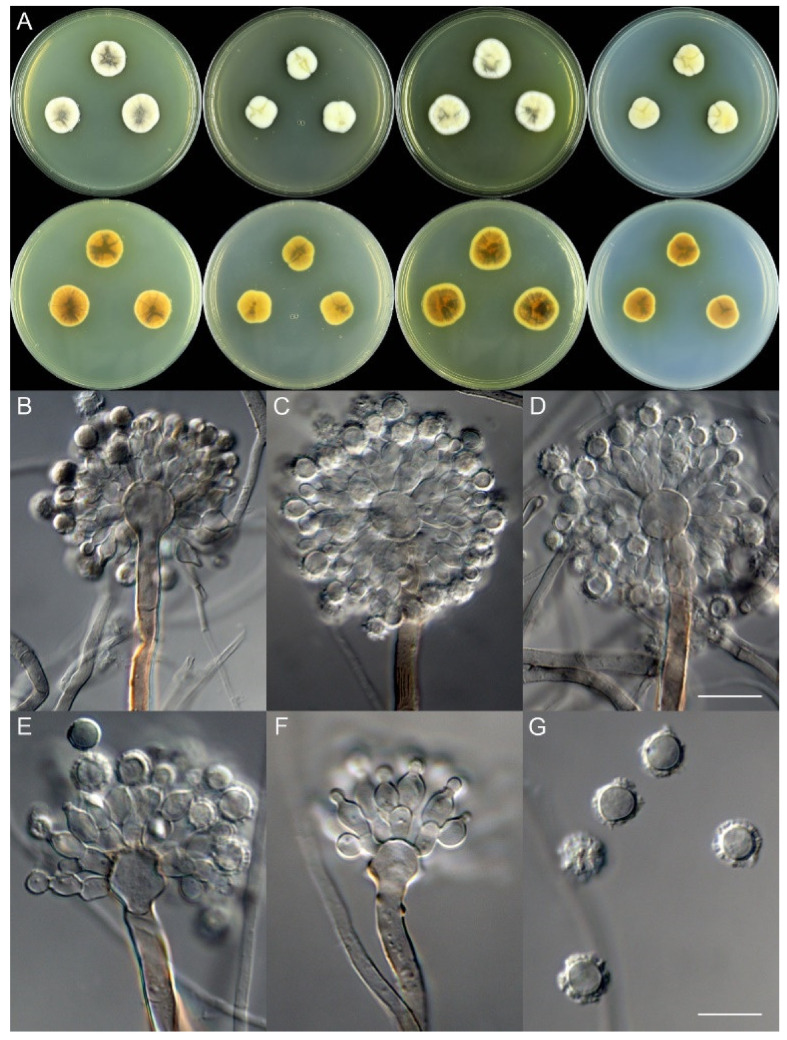
Colonial and microscopic morphology of *Aspergillus hainanicus* (ZC79). (**A**) Colony phenotypes (25 °C, 7 days; top row left to right, obverse CYA, MEA, YES and PDA; bottom row left to right, reverse CYA, MEA, YES and PDA); (**B**–**F**) Conidiophores; (**G**) Conidia. Bars: (**D**) = 15 µm, applies to (**B**,**C**); (**G**) = 10 µm, applies to (**E**,**F**).

***Aspergillus hainanicus*** X.C. Wang and W.Y. Zhuang, sp. nov. Figure 3.

Fungal Names: FN570967.

**Etymology:** The specific epithet refers to the type locality.

In *Aspergillus* subgen. *Nidulantes* sect. *Cavernicolarum* ser. *Hainanici*.

**Typification:** CHINA. Hainan Province, Sansha City, Xisha District, Xisha Islands, Xuande Islands, Yongxing Island, 16°50′4″ N 112°20′49″ E, in sandy soil (phosphorous lime soil) under unidentified plants, 29 March 2015, Ye-Wei Xia, culture, Kai Chen, ZC79 (holotype HMAS 247855, ex-type strain CGMCC 3.20888).

**DNA barcodes:** ITS OM414846, BenA OM475626, CaM OM475630, RPB2 OM475634.

**Colony diam.**: 7 days, 25 °C (unless stated otherwise): CYA 18–20 mm; CYA 37 °C no growth; MEA 16–17 mm; YES 21–22 mm; PDA 16–17 mm.

**Colony characteristics:** On CYA 25 °C, 7 days: Colonies nearly circular, concave at centers, protuberant at margins, radially sulcate; margins narrow, entire; mycelia white and then buff; texture velutinous; sporulation sparse; conidia *en masse* greyish black; soluble pigments light brown; exudates tiny, hyaline and clear; reverse yellow to orange, but black at centers and with black sectors. On MEA 25 °C, 7 days: Colonies irregular, protuberant; margins narrow, entire; mycelia white and then cream to light yellow; texture velutinous; sporulation absent; soluble pigments light brown; exudates tiny, hyaline and clear; reverse buff, yellow to orange, but black at centers. On YES 25 °C, 7 days: Colonies nearly circular or irregular, protuberant at centers, radially sulcate; margins narrow, fimbriate; mycelia white; texture velutinous; sporulation sparse; conidia *en masse* greyish black; soluble pigments greenish-brown; exudates absent; reverse orange to black. On PDA 25 °C, 7 days: Colonies irregular, protuberant; margins narrow, entire; mycelia white and then cream to light yellow; texture velutinous; sporulation absent; soluble pigments yellow; exudates tiny, hyaline and clear; reverse buff, yellow to orange, and with black sectors.

**Micromorphology:** Conidial heads radiate; stipes short, 55–90 × 4.5–6.0 μm, thick walls, smooth, brown, not septate; vesicles 7.5–13 × 9.0–13 μm, globose to subglobose; biseriate; metulae 5.0–9.0 × 3.0–6.5 μm, cylindrical to obovate, covering almost a half surface of the vesicle; phialides 5.5–8.0 × 3.5–5.0 μm, flask-shaped; conidia 6.0–9.5 μm, subglobose, strongly echinulate.

**Note:** This species is phylogenetically related to *A. californicus*, *A. cavernicola*, *A. kassunensis* and *A. subsessilis* of ser. *Cavernicolarum* and *A. egyptiacus* of ser. *Egyptiaci* (Figure 1), but differs from the former four species in its brown stipe and larger, strongly echinulate conidia, and differs from the latter one due to no growth on CYA at 37 °C, slower growth rates on MEA and YES, brown stipe and larger and strongly echinulate conidia (Table 4).

**Figure 4:**
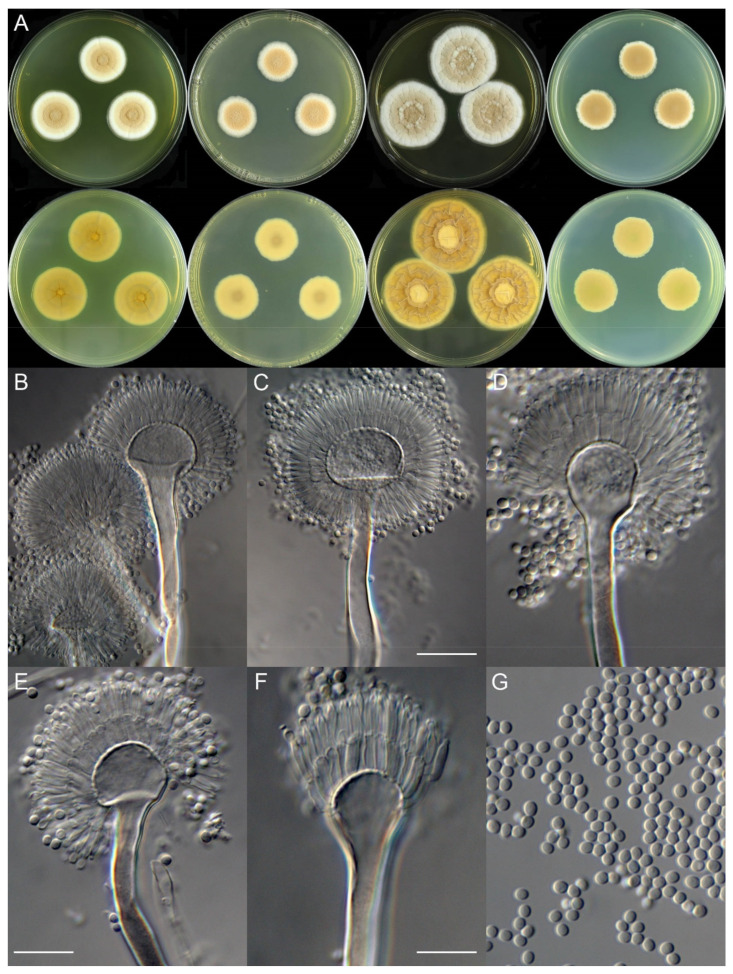
Colonial and microscopic morphology of *Aspergillus neoterreus* (ZC111). (**A**) Colony phenotypes (25 °C, 7 days; top row left to right, obverse CYA, MEA, YES and PDA; bottom row left to right, reverse CYA, MEA, YES and PDA); (**B**–**F**) Conidiophores; (**G**) Conidia. Bars: (**C**) = 20 µm, applies to (**B**); (**E**) = 12.5 µm, applies to (**D**); (**F**) = 10 µm, applies to (**G**).

***Aspergillus neoterreus*** X.C. Wang and W.Y. Zhuang, sp. Nov. Figure 4.

Fungal Names: FN570968.

**Etymology:** The specific epithet refers to the close relationship with *A. terreus*.

In *Aspergillus* subgen. *Circumdati* sect. *Terrei* ser. *Terrei*.

**Typification:** CHINA. Hainan Province, Sansha City, Xisha District, Xisha Islands, Xuande Islands, Qilianyu Islands, Nanshazhou Island, 16°55′46″ N 112°20′55″ E, in sandy soil (phosphorous lime soil) under unidentified plants, 29 March 2015, Ye-Wei Xia, culture, Kai Chen, ZC111 (holotype HMAS 247856, ex-type strain CGMCC 3.20891).

**DNA barcodes:** ITS OM414849, BenA OM475629, CaM OM475633, RPB2 OM475637.

**Colony diam.**: 7 days, 25 °C (unless stated otherwise): CYA 26–28 mm; CYA 37 °C 57–58 mm; MEA 21–23 mm; YES 37–40 mm; PDA 20–22 mm.

**Colony characteristics:** On CYA 25 °C, 7 days: Colonies nearly circular, slightly protuberant at centers, concentrically sulcate; margins narrow, entire; mycelia white; texture velutinous; sporulation moderately dense; conidia *en masse* wheat, yellow-brown to khaki; soluble pigments absent; exudates absent; reverse light brown. On CYA 37 °C, 7 days: Colonies nearly circular or irregular, plain, radially sulcate; margins moderately wide, irregular; mycelia white; texture velutinous; sporulation dense; conidia *en masse* wheat, yellow-brown to khaki; soluble pigments absent; exudates absent; reverse yellow-brown to dark brown. On MEA 25 °C, 7 days: Colonies nearly circular, plain, slightly protuberant at centers; margins wide, entire; mycelia white; texture velutinous; sporulation moderately dense; conidia *en masse* wheat, yellow-brown to khaki; soluble pigments absent; exudates absent; reverse buff to yellow-brown, but light brown at centers. On YES 25 °C, 7 days: Colonies nearly circular, concave at centers, strongly sulcate; margins wide, fimbriate; mycelia white; texture velutinous; sporulation moderately dense; conidia *en masse* wheat, yellow-brown to khaki; soluble pigments absent; exudates absent; reverse yellow-brown to light brown. On PDA 25 °C, 7 days: Colonies nearly circular, plain, slightly protuberant at centers; margins narrow, irregular; mycelia white; texture velutinous; sporulation dense; conidia *en masse* wheat, yellow-brown to khaki; soluble pigments absent; exudates absent; reverse pink-brown, but greenish-brown at centers.

**Micromorphology:** Conidial heads radiate; stipes 150–225 × 2.5–7.5 μm, thick walls, smooth, hyaline or blackish, not septate; vesicles 11–16.5 × 8.5–27 μm, subglobose to ellipsoid; biseriate; metulae 6.0–7.5 × 2.0–3.0 μm, cylindrical, covering a half to two-thirds the surface of the vesicle; phialides 7.0–8.5 × 1.5–2.0 μm, acerose; conidia 2.0–2.5 μm, subglobose to broad ellipsoid, smooth.

**Note:** This species is phylogenetically related to *A. citrinoterreus* (Figure 2) but differs in slower growth rate on CYA and smaller conidia (Table 4).

**Figure 5:**
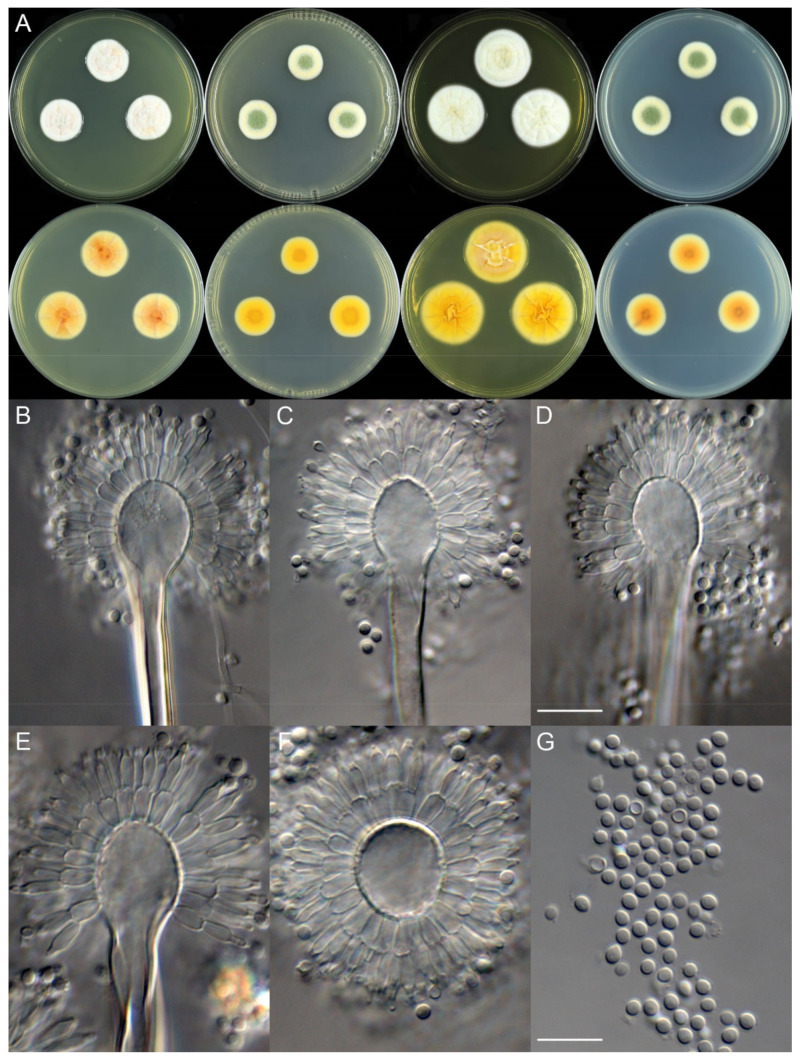
Colonial and microscopic morphology of *Aspergillus qilianyuensis* (ZC101). (**A**) Colony phenotypes (25 °C, 7 days; top row left to right, obverse CYA, MEA, YES and PDA; bottom row left to right, reverse CYA, MEA, YES and PDA); (**B**–**F**) Conidiophores; (**G**) Conidia. Bars: (**D**) = 12.5 µm, applies to (**B**,**C**); (**G**) = 10 µm, applies to (**E**,**F**).

***Aspergillus qilianyuensis*** X.C. Wang and W.Y. Zhuang, sp. Nov. Figure 5.

Fungal Names: FN570969.

**Etymology:** The specific epithet refers to the type locality.

In *Aspergillus* subgen. *Nidulantes* sect. *Nidulantes* ser. *Versicolores*.

**Typification:** CHINA. Hainan Province, Sansha City, Xisha District, Xisha Islands, Xuande Islands, Qilianyu Islands, Nanshazhou Island, 16°55′46″ N 112°20′55″ E, in sandy soil (phosphorous lime soil) under unidentified plants, 29 March 2015, Ye-Wei Xia, culture, Kai Chen, ZC101 (holotype HMAS 247857, ex-type strain CGMCC 3.20889).

**DNA barcodes:** ITS OM414847, BenA OM475627, CaM OM475631, RPB2 OM475635.

**Colony diam.**: 7 days, 25 °C (unless stated otherwise): CYA 21–23 mm; CYA 37 °C no growth; MEA 17–20 mm; YES 29–30 mm; PDA 19–20 mm.

**Colony characteristics:** On CYA 25 °C, 7 days: Colonies nearly circular, protuberant, concentrically and radially sulcate; margins narrow, entire; mycelia white and then pink; texture velutinous; sporulation sparse; conidia *en masse* light greyish green; soluble pigments absent; exudates absent; reverse buff to pink-brown. On MEA 25 °C, 7 days: Colonies nearly circular, slightly protuberant at central areas; margins wide, entire; mycelia white and becoming yellow; texture velutinous; sporulation moderately dense; conidia *en masse* greyish green; soluble pigments absent; exudates absent; reverse buff to vivid yellow, but orange-brown at centers. On YES 25 °C, 7 days: Colonies nearly circular, protuberant or concave at centers, concentrically and radially sulcate, deep; margins narrow, entire; mycelia white; texture velutinous; sporulation sparse; conidia *en masse* light yellow; soluble pigments absent; exudates absent; reverse yellow-brown. On PDA 25 °C, 7 days: Colonies nearly circular, slightly protuberant at central areas; margins wide, entire; mycelia white and then yellow; texture velutinous; sporulation moderately dense; conidia *en masse* greyish green; soluble pigments absent; exudates absent; reverse buff, yellow-brown to orange-brown.

**Micromorphology:** Conidial heads radiate; stipes 225–325 × 4.0–8.0 μm, thick walls, smooth, hyaline or blackish, not septate; vesicles 16–20 × 10–18 μm, ellipsoid; biseriate; metulae 5.0–6.0 × 3.0–3.5 μm, cylindrical, covering two-thirds to almost the entire surface of the vesicle; phialides 6.0–8.0 × 2.0–2.5 μm, flask-shaped to acerose; conidia 2.0–3.0 μm, subglobose, smooth.

**Note:** This species formed a distinct lineage in ser. *Versicolores* (Figure 1). Morphologically, it differs from the type species of this series, *A. versicolor*, in slower growth rates on CYA and MEA and smooth and smaller conidia (Table 4).

**Figure 6:**
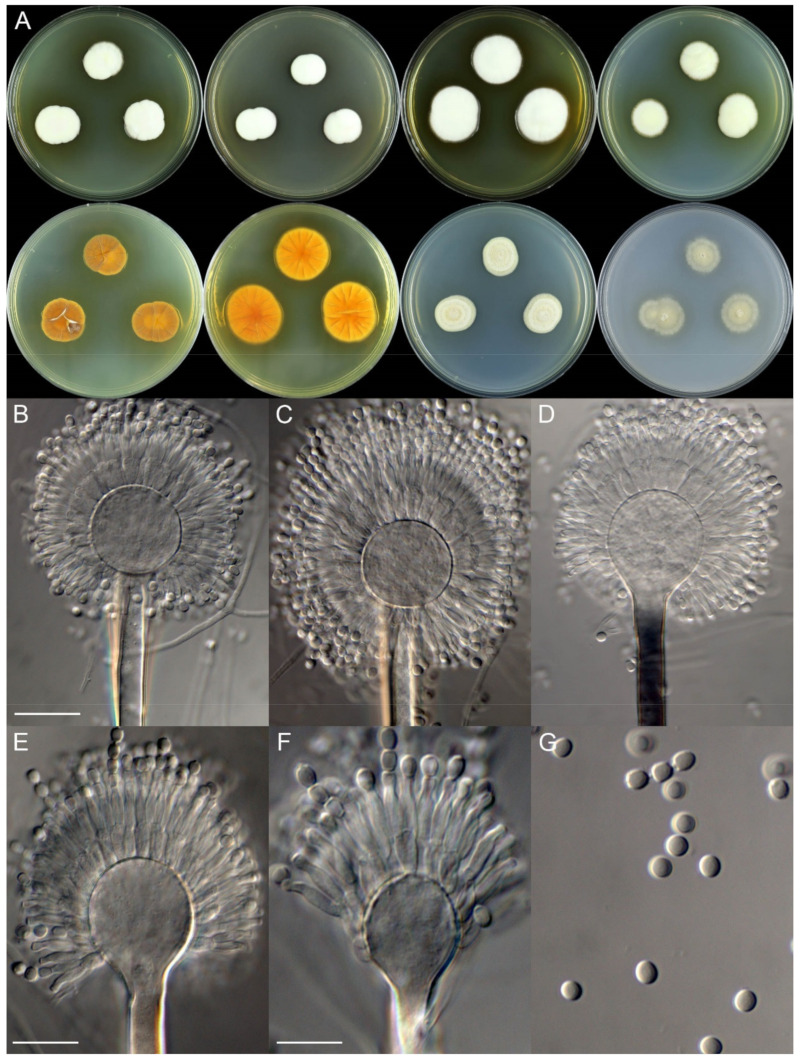
Colonial and microscopic morphology of *Aspergillus xishaensis* (ZC108). (**A**) Colony phenotypes (25 °C, 7 days; top row left to right, obverse CYA, MEA, YES and PDA; bottom row left to right, reverse CYA and YES, obverse PDA with 3% NaCl and OA); (**B**–**F**) Conidiophores; (**G**) Conidia. Bars: (**B**) = 20 µm, applies to (**C**,**D**); (**E**) = 15 µm; (**F**) = 10 µm, applies to (**G**).

***Aspergillus xishaensis*** X.C. Wang and W.Y. Zhuang, sp. nov. Figure 6.

Fungal Names: FN570970.

**Etymology:** The specific epithet refers to the type locality.

In *Aspergillus* subgen. *Circumdati* sect. *Flavipedes* ser. *Flavipedes*.

**Typification:** CHINA. Hainan Province, Sansha City, Xisha District, Xisha Islands, Xuande Islands, Qilianyu Islands, Nanshazhou Island, 16°55′46″ N 112°20′55″ E, in sandy soil (phosphorous lime soil) under unidentified plants, 29 March 2015, Ye-Wei Xia, culture, Kai Chen, ZC108 (holotype HMAS 247858, ex-type strain CGMCC 3.20890).

**DNA barcodes:** ITS OM414848, BenA OM475628, CaM OM475632, RPB2 OM475636.

**Colony diam.**: 7 days, 25 °C (unless stated otherwise): CYA 19–22 mm; CYA 37 °C 19–21 mm; MEA 16–20 mm; YES 25–29 mm; PDA 18–22 mm; PDA (3% NaCl) 19–20 mm; OA 19–20 mm.

**Colony characteristics:** On CYA 25 °C, 7 days: Colonies irregular, protuberant, radially sulcate; margins narrow, entire; mycelia white and then light yellow; texture velutinous; sporulation absent; soluble pigments yellow-brown; exudates absent; reverse yellow-brown to light brown. On CYA 37 °C, 7 days: Colonies nearly circular, protuberant at centers, radially sulcate; margins wide, fimbriate; mycelia white and then light yellow; texture velutinous; sporulation absent; soluble pigments yellow-brown; exudates absent; reverse yellow-brown to dark brown. On MEA 25 °C, 7 days: Colonies nearly circular or irregular, protuberant; margins narrow, entire; mycelia white and then cream; texture velutinous; sporulation absent; soluble pigments yellow-brown; exudates absent; reverse light brown, but buff at margins. On YES 25 °C, 7 days: Colonies nearly circular, protuberant at centers, radially sulcate; margins narrow, entire; mycelia white and then light cream; texture velutinous; sporulation absent; soluble pigments yellow-brown; exudates absent; reverse yellow-brown to orange-brown. On PDA 25 °C, 7 days: Colonies nearly circular or irregular, protuberant; margins narrow, entire; mycelia white and then light cream; texture velutinous; sporulation absent; soluble pigments yellow-brown; exudates greenish-yellow, clear; reverse yellow-brown to light brown. On PDA (3% NaCl) 25 °C, 7 days: Colonies oblong, protuberant; margins moderately wide, entire; mycelia cream; texture velutinous; sporulation dense; conidia *en masse* white to cream; soluble pigments light yellow-brown; exudates absent; reverse yellow-brown to light brown. On OA 25 °C, 7 days: Colonies nearly circular or irregular, protuberant; margins wide, fimbriate; mycelia cream; texture velutinous; sporulation sparse; conidia *en masse* white to cream; soluble pigments yellow-brown; exudates absent; reverse light yellow, but light brown at centers.

**Micromorphology:** Conidial heads radiate; stipes long, 700–1400 × 7.5–10 μm, thick walls, smooth, hyaline or blackish, not septate; vesicles 18–35 × 15–35 μm, globose to broad ellipsoid; biseriate; metulae 7.0–11 × 3.5–4.5 μm, cylindrical, covering two thirds to almost the entire surface of the vesicle; phialides 9.0–11.5 × 2.5–3.0 μm, flask-shaped to acerose; conidia 3.0–4.0 μm, globose to subglobose, smooth.

**Note:** This species is phylogenetically related to *A. micronesiensis* and *A. neoflavipes* (Figure 2) but differs from them in slower growth rates on CYA, MEA and YES and larger conidia (Table 4).

## 4. Discussion

*Aspergillus* is a large genus with more than 400 accepted species and more than 1000 names. A comprehensive taxonomic treatment of the genus was recently established on the basis of molecular data and morphological characteristics [6]. Six subgenera, twenty-seven sections and seventy-five series were currently accepted, among which five new sections and seventy-three new series were erected. Based on the above treatment, researchers are able to quickly position their materials to specific ranks of series, sections and subgenera. Three of the four new species described in this study were classified into the known series, except for *A. hainanicus*, for which the new series *Hainanici* is proposed. Along with future discovery of new taxa, the current classification system may be updated.

In the *Flavi*, *Fumigati*, *Nigri* and *Terrei* sections of *Aspergillus*, some species cause the infectious disease aspergillosis, such as the most frequently occurred and well-known pathogen *A. fumigatus* [36]. In sect. *Nidulantes*, *A. versicolor* (Vuill.) Tirab., a close relative of *A. qilianyuensis*, was isolated from the skin [37] and nails [38] of humans and also invasively infected multiple organs of dogs [39,40]. *Aspergillus hongkongensis* C.C. Tsang et al. causes onychomycosis [41]. In subgen. *Circumdati*, *A. citrinoterreus* J. Guinea et al. and *A. suttoniae* J.P.Z. Siqueira et al. were isolated from the sputum of humans [35,42], and *A. alabamensis* Balajee et al. from the wounds of humans [43]. Whether others of these sections are potentially pathogenic requires future investigation.

Tropical islands represent a unique ecosystem. Due to their extremely isolated location and special environmental conditions, some of them are considered as the world’s biodiversity hotspots. Several species of *Aspergillus* were recorded from similar geographical origins, such as *A. griseoaurantiacus* Visagie et al. and *A. micronesiensis* Visagie et al. from Micronesia [33], and *A. puulaauensis* Jurjević et al. from Hawaii [32]. The four new species were all derived from the soil samples of the Xisha Islands, which seem to exhibit high species diversity. Further explorations on tropical islands are desperately needed, and we certainly expect to find more new fungi there.

## Figures and Tables

**Table 1:** Fungal species and sequences used in phylogenetic analyses of *Aspergillus* subgen. *Nidulantes*.

Section	Series	Species	Strain	Locality	Substrate	ITS	BenA	CaM	RPB2
*Aenei*	*Aenei*	*A. aeneus* Sappa 1954	CBS 128.54 T	Somalia	forest soil	EF652474	EF652298	EF652386	EF652210
		*A. bicolor* M. Chr. and States 1978	CBS 425.77 T	USA	soil	EF652511	EF652335	EF652423	EF652247
*Bispori*	*Bispori*	*A. bisporus* Kwon-Chung and Fennell 1971	CBS 707.71 T	USA	soil	EF661208	EF661121	EF661139	EF661077
*Cavernicolarum*	*Cavernicolarum*	*A. californicus* Frisvad et al. 2011	CBS 123895 T	USA	chaparral of *Adenostoma fasciculatum*	FJ531153	FJ531180	FJ531128	MN969065
		*A. cavernicola* Lörinczi 1969	CBS 117.76 T	Romania	on walls of cave	EF652508	EF652332	EF652420	EF652244
		*A. kassunensis* Baghd. 1968	CBS 419.69 T	Syria	soil	EF652461	EF652285	EF652373	EF652197
		*A. subsessilis* Raper and Fennell 1965	CBS 502.65 T	USA	desert soil	EF652485	EF652309	EF652397	EF652221
	*Egyptiaci*	*A. egyptiacus* Moub. and Moustafa 1972	CBS 656.73 T	Egypt	sandy soil	EF652504	EF652328	EF652416	EF652240
	***Hainanici***	***A. hainanicus*** X.C. Wang and W.Y. Zhuang, sp. nov.	ZC79 T	China: Hainan	sandy soil	**OM414846**	**OM475626**	**OM475630**	**OM475634**
*Nidulantes*	*Aurantiobrunnei*	*A. aurantiobrunneus* Raper and Fennell 1965	CBS 465.65 T	Australia	canvas haversack for respirator	EF652465	EF652289	EF652377	EF652201
	*Multicolores*	*A. multicolor* Sappa 1954	CBS 133.54 T	Somalia	forest soil	EF652477	EF652301	EF652389	EF652213
	*Nidulantes*	*A. nidulans* (Eidam) G. Winter 1884	CBS 589.65 T	Belgium	unknown	EF652427	EF652251	EF652339	EF652163
	*Speluncei*	*A. spelunceus* Raper and Fennell 1965	CBS 497.65 T	USA	soil and dead *Orthoptera*	EF652490	EF652314	EF652402	EF652226
	*Stellati*	*A. stellatus* Curzi 1934	CBS 598.65 T	Panama	soil	EF652426	EF652250	EF652338	EF652162
	*Unguium*	*A. unguis* (Émile-Weill and L. Gaudin) Thom and Raper 1934	CBS 132.55 T	USA	shoe leather	EF652443	EF652267	EF652355	EF652179
	*Versicolores*	*A. amoenus* M. Roberg 1931	CBS 111.32 T	Germany	fruit of *Berberis* sp.	EF652480	JN853946	JN854035	JN853824
		*A. austroafricanus* Jurjević et al. 2012	CBS 145748 T	South Africa	soil	JQ301891	JN853963	JN854025	JN853814
		*A. creber* Jurjević et al. 2012	CBS 145749 T	USA	air	JQ301889	JN853980	JN854043	JN853832
		*A. cvjetkovicii* Jurjević et al. 2012	CBS 599.65 T	USA	soil	EF652440	EF652264	EF652352	EF652176
		*A. fructus* Jurjević et al. 2012	CBS 584.65 T	USA	fruit of date palm	EF652449	EF652273	EF652361	EF652185
		*A. griseoaurantiacus* Visagie et al. 2014	CBS 138191 T	Micronesia	house dust	KJ775553	KJ775086	KJ775357	KU866988
		*A. hongkongensis* C.C. Tsang et al. 2016	CBS 145671 T	China: Hong Kong	nails of *Homo sapiens*	AB987907	LC000552	MN969320	LC000578
		*A. jensenii* Jurjević et al. 2012	NRRL 58600 T	USA	soil	JQ301892	JN854007	JN854046	JN853835
		*A. pepii* Despot et al. 2016	CBS 142028 T	Croatia	air	KU613368	KU613371	KU613365	n.a.
		*A. protuberus* Munt.-Cvetk. 1968	CBS 602.74 T	former Yugoslavia	rubber coated electric cables	EF652460	EF652284	EF652372	EF652196
		*A. puulaauensis* Jurjević et al. 2012	CBS 145750 T	USA: Hawaii	dead hardwood	JQ301893	JN853979	JN854034	JN853823
		***A. qilianyuensis*** X.C. Wang and W.Y. Zhuang, sp. nov.	ZC101 T	China: Hainan	sandy soil	**OM414847**	**OM475627**	**OM475631**	**OM475635**
		*A. subversicolor* Jurjević et al. 2012	CBS 145751 T	India	green berries of coffee	JQ301894	JN853970	JN854010	JN853799
		*A. sydowii* (Bainier and Sartory) Thom and Church 1926	CBS 593.65 T	France	unknown	EF652450	EF652274	EF652362	EF652186
		*A. tabacinus* Nakaz. et al. 1934	CBS 122718 T	unknown	tobacco	EF652478	EF652302	EF652390	EF652214
		*A. tennesseensis* Jurjević et al. 2012	CBS 145752 T	USA	toxic dairy feed	JQ301895	JN853976	JN854017	JN853806
		*A. venenatus* Jurjević et al. 2012	CBS 145753 T	USA	toxic dairy feed	JQ301896	JN854003	JN854014	JN853803
		*A. versicolor* (Vuill.) Tirab. 1908	CBS 583.65 T	unknown	unknown	EF652442	EF652266	EF652354	EF652178
*Ochraceorosei*	*Funiculosi*	*A. funiculosus* G. Sm. 1956	NRRL 4744 T	Nigeria	loam soil	EF661223	EF661112	EF661175	EF661078
		*A. lannaensis* N. Suwannarach et al. 2021	SDBR-CMUO8 T	Thailand	soil	MW588211	MW219783	MW219781	MW219785
	*Ochraceorosei*	*A. ochraceoroseus* Bartoli and Maggi 1979	CBS 550.77 T	Côte d’Ivoire	forest soil	EF661224	EF661113	EF661137	EF661074
*Raperorum*	*Raperorum*	*A. ivoriensis* Bartoli and Maggi 1979	CBS 551.77 T	Côte d’Ivoire	forest soil	EF652441	EF652265	EF652353	EF652177
		*A. raperi* Stolk and J.A. Mey. 1957	CBS 123.56 T	Congo	soil	EF652454	EF652278	EF652366	EF652190
*Silvatici*	*Silvatici*	*A. silvaticus* Fennell and Raper 1955	CBS 128.55 T	Ghana	soil	EF652448	EF652272	EF652360	EF652184
Sparsi	*Biplani*	*A. biplanus* Raper and Fennell 1965	CBS 468.65 T	Costa Rica	soil	EF661210	EF661116	EF661130	EF661036
	*Conjuncti*	*A. conjunctus* Kwon-Chung and Fennell 1965	CBS 476.65 T	Costa Rica	soil	EF661179	EF661111	EF661133	EF661042
	*Implicati*	*A. implicatus* Persiani and Maggi 1994	CBS 484.95 T	Côte d’Ivoire	forest soil	FJ491656	FJ491667	FJ491650	MN969078
	*Sparsi*	*A. sparsus* Raper and Thom 1944	CBS 139.61 T	Costa Rica	soil	EF661181	EF661125	EF661173	EF661071
Usti	*Calidousti*	*A. calidoustus* Varga et al. 2008	CBS 121601 T	Netherlands	bronchoalveolar lavage fluid of *Homo sapiens*	HE616558	FJ624456	HE616559	MN969061
	*Deflecti*	*A. deflectus* Fennell and Raper 1955	CBS 109.55 T	Brazil	soil	EF652437	EF652261	EF652349	EF652173
	*Monodiorum*	*A. monodii* (Locq.-Lin.) Varga et al. 2011	CBS 435.93 T	Chad	dung of *Agnus*	FJ531150	FJ531171	FJ531142	MN969082
	*Usti*	*A. ustus* (Bainier) Thom and Church 1926	CBS 261.67 T	USA	culture contaminant	EF652455	EF652279	EF652367	EF652191
outgroup		*A. flavus* Link 1809	CBS 569.65 T	South Pacific	cellophane	AF027863	EF661485	EF661508	EF661440

GenBank accession numbers in bold indicate the newly generated sequences.

**Table 2:** Fungal species and sequences used in phylogenetic analyses of *Aspergillus* subgen. *Circumdati*.

Section	Series	Species	Strain	Locality	Substrate	ITS	BenA	CaM	RPB2
*Flavipedes*	*Flavipedes*	*A. ardalensis* A. Nováková et al. 2015	CBS 134372 T	Spain	soil	FR733808	HG916683	HG916725	HG916704
		*A. capensis* Visagie et al. 2014	CBS 138188 T	South Africa	house dust	KJ775550	KJ775072	KJ775279	KP987020
		*A. flavipes* (Bainier and R. Sartory) Thom and Church 1926	NRRL 302 T	France	dung of dog	EF669591	EU014085	EF669549	EF669633
		*A. iizukae* Sugiy 1967	CBS 541.69 T	Japan	core sample from stratigraphic drilling	EF669597	EU014086	EF669555	EF669639
		*A. micronesiensis* Visagie et al. 2014	CBS 138183 T	Micronesia	house dust	KJ775548	KJ775085	KP987067	KP987023
		*A. neoflavipes* Hubka et al. 2015	CBS 260.73 T	Thailand	forest soil	EF669614	EU014084	EF669572	EF669656
		*A. okavangoensis* Visagie and Nkwe 2021	CBS 147420 T	Botswana	bat guano contaminated soil in cave	MW480880	MW480788	MW480706	MW480790
		*A. suttoniae* J.P.Z. Siqueira et al. 2018	FMR 13523 T	USA	sputum of *Homo sapiens*	LT899487	LT899536	LT899589	LT899644
		*A. templicola* Visagie et al. 2014	CBS 138181 T	Mexico	church dust	KJ775545	KJ775092	KJ775394	KP987017
		*A. urmiensis* Arzanlou et al. 2016	CBS 139558 T	Iran	soil	KP987073	KP987041	KP987056	KP987030
		***A. xishaensis*** X.C. Wang and W.Y. Zhuang, sp. nov.	ZC108 T	China: Hainan	sandy soil	**OM414848**	**OM475628**	**OM475632**	**OM475636**
*Terrei*	*Terrei*	*A. alabamensis* Balajee et al. 2009	CBS 125693 T	USA	wound of *Homo sapiens*	KP987071	KP987049	EU147583	KP987018
		*A. aureoterreus* Samson et al. 2011	CBS 503.65 T	USA	soil	EF669580	EF669524	EF669538	EF669622
		*A. citrinoterreus* J. Guinea et al. 2015	CBS 138921 T	Spain	sputum of *Homo sapiens*	KP175260	LN680657	LN680685	MN969155
		*A. floccosus* (Y.K. Shih) Samson et al. 2011	CBS 116.37 T	China: Hubei	waste cloth	KP987086	FJ491714	KP987066	KP987021
		*A. heldtiae* Visagie 2020	PPRI 4229 T	South Africa	seed of *Pennisetum glaucum*	MK450656	MK450981	MK451518	MK450809
		*A. hortae* (Langeron) C.W. Dodge 1935	CBS 124230 T	Brazil	ear of *Homo sapiens*	KP987087	FJ491706	KP987054	KP987022
		*A. neoafricanus* Samson et al. 2011	CBS 130.55 T	Ghana	soil	EF669585	EF669516	EF669543	EF669627
		***A. neoterreus*** X.C. Wang and W.Y. Zhuang, sp. nov.	ZC111 T	China: Hainan	sandy soil	**OM414849**	**OM475629**	**OM475633**	**OM475637**
		*A. pseudoterreus* S.W. Peterson et al. 2011	CBS 123890 T	Argentina	soil	EF669598	EF669523	EF669556	EF669640
		*A. terreus* Thom 1918	CBS 601.65 T	USA	soil	EF669586	EF669519	EF669544	EF669628
*Flavi*	*Flavi*	*A. flavus* Link 1809	CBS 569.65 T	South Pacific	cellophane	AF027863	EF661485	EF661508	EF661440

GenBank accession numbers in bold indicate the newly generated sequences.

**Table 3:** Detailed characteristics of datasets of *Aspergillus*.

Subgenus	Locus	No. of Seq.	Length of Alignment (bp)	No. of Variable Sites	No. of Parsimony-Informative Sites	Model for BI
Nidulantes	BenA	48	528	292	235	
	CaM	48	829	477	408	
	RPB2	47	1014	429	377	
	combined	48	2371	1198	1020	TIM + I + G
Circumdati	BenA	22	541	273	199	
	CaM	22	589	286	218	
	RPB2	22	998	301	216	
	combined	22	2128	860	633	TIM + I + G

Full names of the used models: TIM + I + G (transition model with invariable sites and gamma distribution).

**Table 4:** Morphological comparisons of new species and their closely related species.

Species	CYA 25 °C (mm)	CYA 37 °C (mm)	MEA (mm)	YES (mm)	Conidia Shape	Conidia Wall	Conidia Size (µm)	Reference
*A. cavernicola*	10–12	no growth	12–13	14–15	subglobose	smooth to echinulate	5–6 × 3.5–4.5	[19]
*A. californicus*	20–24	no growth	19–20	26–27	subglobose to ellipsoidal	smooth to finely roughened	3–4.5 × 2.5–4.5	[19]
*A. kassunensis*	15–16	no growth	18–20	21–22	globose	smooth	2–3	[19]
*A. subsessilis*	16–17	no growth	12–13	18–19	globose	smooth	3–4	[19]
*A. egyptiacus*	13–20	21–24	29–30	32–45	globose to subglobose	smooth	4.5–6.5 × 3.5–6	[19]
*A. hainanicus*	18–20	no growth	16–17	21–22	subglobose	strongly echinulate	6–9.5	This study
*A. versicolor*	28–36	8	21–31	n.a.	spherical to subspherical	finely roughened	2.5–3.5	[32]
*A. qilianyuensis*	21–23	no growth	17–20	29–30	subglobose	smooth	2–3	This study
*A. micronesiensis*	22–28	17–25	20–25	35–44	globose to subglobose	smooth to finely roughened	2.5–3.5	[33]
*A. neoflavipes*	30–33	20–22	34–35	n.a.	globose to subglobose	smooth	2.5–3	[34]
*A. xishaensis*	19–22	19–21	16–20	25–29	globose to subglobose	smooth	3–4	This study
*A. citrinoterreus*	33–35	n.a.	23–25	n.a.	globose to subglobose	smooth	2–3 × 1.5–3	[35]
*A. neoterreus*	26–28	57–58	21–23	37–40	subglobose to broad ellipsoid	smooth	2–2.5	This study

## Data Availability

The sequences newly generated in this study have been submitted to the GenBank database.
